# Interleukin 10 knock-down in bovine monocyte-derived macrophages has distinct effects during infection with two divergent strains of *Mycobacterium bovis*

**DOI:** 10.1371/journal.pone.0222437

**Published:** 2019-09-17

**Authors:** Kirsty Jensen, Joanne M. Stevens, Elizabeth J. Glass

**Affiliations:** Division of Infection & Immunity, The Roslin Institute & Royal (Dick) School of Veterinary Studies, University of Edinburgh, Midlothian, United Kingdom; University of British Columbia, CANADA

## Abstract

*Mycobacterium bovis* is the causative agent of bovine tuberculosis (TB), a cattle disease of global importance. *M*. *bovis* infects bovine macrophages (Mø) and subverts the host cell response to generate a suitable niche for survival and replication. We investigated the role of the anti-inflammatory cytokine interleukin (IL) 10 during *in vitro* infection of bovine monocyte-derived Mø (bMDM) with two divergent UK strains of *M*. *bovis*, which differentially modulate expression of IL10. The use of IL10-targeting siRNA revealed that IL10 inhibited the production of IL1B, IL6, tumour necrosis factor (TNF) and interferon gamma (IFNG) during infection of bMDM with the *M*. *bovis* strain G18. In contrast, IL10 only regulated a subset of these genes; TNF and IFNG, during infection with the *M*. *bovis* reference strain AF2122/97. Furthermore, nitric oxide (NO) production was modulated by IL10 during AF2122/97 infection, but not at the nitric oxide synthase 2 (NOS2) mRNA level, as observed during G18 infection. However, IL10 was found to promote survival of both *M*. *bovis* strains during early bMDM infection, but this effect disappeared after 24 h. The role of IL10-induced modulation of TNF, IFNG and NO production in *M*. *bovis* survival was investigated using siRNA targeting TNF, IFNG receptor 1 (IFNGR1) and NOS2. Knock-down of these genes individually did not promote survival of either *M*. *bovis* strain and therefore modulation of these genes does not account for the effect of IL10 on *M*. *bovis* survival. However, TNF knock-down was found to be detrimental to the survival of the *M*. *bovis* strain G18 during early infection. The results provide further evidence for the importance of IL10 during *M*. *bovis* infection of Mø. Furthermore, they highlight *M*. *bovis* strain specific differences in the interaction with the infected bMDM, which may influence the course of infection and progression of bovine TB.

## Introduction

*Mycobacterium bovis* is the causative agent of bovine tuberculosis (TB), which is an important disease of cattle and other animals around the globe. *M*. *bovis* is closely related to *Mycobacterium tuberculos*is, the aetiological agent of human TB. Similar to human disease, bovine TB results from inhalation of infectious mycobacteria into the lungs, where the pathogen is phagocytozed by alveolar macrophages (Mø). Mycobacteria utilize a range of mechanisms to subvert the ability of the host cell to kill the invading pathogen (reviewed by [1]). This allows mycobacteria to persist within the infected cell, resulting in the formation of granuloma, aggregates of immune cells that surround and enclose the infected cells, which are indicative of mycobacteria infection.

Although a test-and-slaughter policy has been in place in the United Kingdom (Great Britain and Northern Ireland) for over fifty years, the incidence of bovine TB has increased in recent years, with over 44,000 skin-test positive animals being slaughtered in Great Britain in 2018 [2]. The role of various factors in the increase of bovine TB has been investigated, e.g. environmental factors, host genetics, farm management practices (reviewed by [3]). In contrast, the potential role of the genetics of the pathogen has received less attention. This may be due to the perceived lack of genetic variation amongst UK *M*. *bovis* strains, which exhibit less genetic diversity than the smaller population of *M*. *bovis* in France [4]. However, strains circulating in Northern Ireland exhibit differences in virulence, determined by the proportion of skin-test positive animals which had visible granulomas [5]. Furthermore, we have shown previously that two strains of *M*. *bovis* isolated in the UK interact in significantly different ways with bovine monocyte derived Mø (bMDM), a model for alveolar Mø [6], including variation in intracellular mycobacterial survival and replication, host cell survival and the transcriptional response of bMDM induced by infection. These differences may alter the formation of granuloma, which in turn may impact on disease progression.

The mechanisms behind the dissimilar bMDM response to the two *M*. *bovis* strains are unknown. Infection with one *M*. *bovis* strain, AF2122/97 induced a much greater transcriptional response by bMDM, with respect to the number of differentially expressed genes and the level of differential expression [6]. Only approximately ten percent of genes were affected to a greater extent by the second *M*. *bovis* strain, G18. AF2122/97 also induced more host cell death and proliferated more rapidly in bMDM than G18. However, from the comparison of just two strains it is not possible to determine whether the broad variation in the bMDM response is typical for UK *M*. *bovis* strains or if AF2122/97 and G18 are hyper-stimulatory or hypo-stimulatory, respectively. Furthermore, it is unclear if G18 is actively dampening down the response of the infected bMDM to promote infection.

One gene that was found to be expressed by bMDM at significantly higher levels during G18 infection than AF2122/97 infection was interleukin (IL) 10. IL10 is a pleiotropic cytokine that acts as a master regulator of the immune system, preventing inflammatory pathology by limiting the immune response. Many pathogens, including viruses, bacteria and protozoa, have been shown to manipulate IL10 signalling to promote infection (reviewed by [7]). IL10 has previously been shown to promote the intramacrophage survival of several species of mycobacteria; including *M*. *tuberculosis* survival in human Mø [8] and *Mycobacterium avium* subspecies *paratuberculosis* (MAP) [9] and *M*. *bovis* survival in bMDM [10]. *M*. *tuberculosis* and MAP induce and enhance the production of IL10 at the transcriptional and post-transcriptional level [11, 12]. Pre-treatment of bMDM with neutralizing anti-IL10 antibody enhanced the response of bMDM to MAP infection; increasing nitric oxide (NO) production, phagosome acidification, apoptosis and production of pro-inflammatory cytokines, e.g. tumour necrosis factor (TNF) (formally known as tumour necrosis factor alpha) and IL12 [9]. Therefore, since the *M*. *bovis* strain G18 induced bMDM to produce higher levels of IL10 than AF2122/97, we hypothesized that IL10 may be involved in dampening down the response of bMDM to G18 and be more important in promoting G18 intracellular survival than AF2122/97. We investigated this by attempting to answer a series of questions. Firstly, what is the mechanism behind the strain-specific differential expression of IL10? Secondly, is IL10 responsible for dampening down the transcriptional response of bMDM to G18? Thirdly, if IL10 promotes *M*. *bovis* infection of bMDM, is it more important for G18 than AF2122/97? To address these questions, we inhibited IL10 production during *M*. *bovis* infection of bMDM using siRNA. IL10 was found to regulate a subset of the previously identified transcriptional response of bMDM to infection [6], but this varied with the two strains of *M*. *bovis* investigated. Furthermore, although IL10 was differentially expressed by bMDM in response to infection with the two *M*. *bovis* strains, the cytokine promoted the early survival of both strains.

## Materials & methods

### Animals

Peripheral blood mononuclear cells (PBMC) were isolated from female Holstein-Friesian cattle maintained at The University of Edinburgh Langhill farm, which is free from *M*. *bovis* and MAP. All cattle were less than one year old and were clinically normal. All experimental protocols were authorised under the UK Animals (scientific procedures) Act, 1986 under project licence number P803DD07A Immunity and Resistance to Disease in Ruminants. In addition, The Roslin Institute’s Animal Welfare and Ethics Committee (AWEC) ensure compliance with all relevant legislation and promote the adoption and developments of the 3Rs.

### Bacterial strains and culture

bMDM were infected with two strains of *M*. *bovis* isolated in the United Kingdom; AF2122/97 (spoligotype SB0140, lineage A) and G18 (spoligotype SB0129, lineage P). The isolates were cloned prior to growth to mid-log phase (OD_600_ 0.6–0.8) in liquid Middlebrook 7H9 medium supplemented with 10% albumin-dextrose-catalase (ADC) and 0.1% Tween-80. The mycobacteria were then harvested and prepared as single cell cultures before being frozen. The quantity of *M*. *bovis* in the frozen stocks and the proportion of viable mycobacteria were assessed by quantifying and comparing the number of colony forming units (CFU) and genome copy number (GCN) in a sample of the frozen vials.

### Macrophage culture & challenge

bMDM were generated using a protocol similar to that described previously [13]. In brief, PBMC were cultured for 2 h in RPMI-1640 medium without serum at 5 x 10^6^ cells/ml, before the medium was replaced with bMDM medium (RPMI-1640 supplemented with 16.7% FBS, 4mM L-glutamine and 50μM β-mercaptoethanol) with 100U/ml Penicillin-Streptomycin. The bMDM medium was replaced on day 7. On day 11 the adherent cells were rigorously washed with phosphate buffered saline (PBS) and detached with TrypLE Express (Thermo Fisher Scientific). Purified bMDM were resuspended at 3 x 10^5^ cells/ml in bMDM medium without Penicillin-Streptomycin, aliquotted into 12 well plates and cultured for 72 h before infection on day 14.

Frozen mycobacteria stocks were thawed and prepared in bMDM medium and inoculated at a multiplicity of infection (MOI) of 5. Medium only was added to the uninfected controls. Plates were centrifuged at 300 *g* for 5 minutes and the medium was replaced 1 h post infection.

### Quantification of intracellular *M*. *bovis*

The intracellular mycobacterial GCN in each sample was quantified by qPCR as described previously [6]. Briefly, genomic DNA (gDNA) was isolated from bMDM after infection with *M*. *bovis* for 2, 24 and 48 h using the DNeasy Blood & Tissue kit (Qiagen), following the manufacturer’s instructions, including the pre-treatment of samples with lysozyme to maximise recovery of mycobacterial DNA. The abundance of mycobacterial genome copies in each sample was quantified by qPCR by amplification of the *M*. *bovis* 85B antigen gene (MY85B) as described previously [6].

Intracellular mycobacteria were also quantified as CFU. Infected bMDM were lysed 2, 24 and 48 h post infection (hpi) with 0.5 ml PBS + 0.1% Triton-X-100 and incubated at room temperature for 10 minutes. Aliquots of a 10 fold dilution series were plated onto Middlebrook 7H11 agar plates supplemented with 10% oleic-albumin-dextrose-catalase (OADC) and incubated at 37°C until colonies could be accurately counted.

### Knock-down of target genes by siRNA

bMDM were harvested on day 9, resuspended at 3 x 10^5^ cells/ml in bMDM medium, dispensed into 12 well plates and cultured for 72 h before transfection of siRNA. siRNA duplexes were designed and supplied by Sigma-Aldrich (Table 1). The bMDM were transfected using Lipofectamine RNAiMAX (Thermo Fisher Scientific) as described previously [14]. The MISSION® siRNA Universal Negative Control #1 siRNA (Sigma-Aldrich), which does not share homology with any known mammalian gene, was used as a non-target siRNA control (NTC siRNA). After 24 h the medium was replaced with fresh medium to remove the residual siRNA/Lipofectamine RNAiMAX mix. After a further 24 h (day 14) cells were infected with *M*. *bovis* strains as described above or stimulated with 100 ng/ml phenol-extracted lipopolysaccharide (LPS) derived from *Escherichia coli* 055:B5 (Sigma-Aldrich) with or without the addition of 500 pg/ml recombinant bovine IFNG (Bio-Rad).

**Table 1:** Details of the siRNA used in this study.

Gene	Accession No.	Target sequence (5’-3’)
interferon gamma receptor 1 (IFNGR1)	NM_001035063	GAAAGTTGGACCACCTAAA
interleukin 10 (IL10)	NM_174088	CATAGAAACCTACATGACA
nitric oxide synthase 2 (NOS2/iNOS)	NM_001076799	GCTATTGGGTCAAGGATAA
tumour necrosis factor (TNF)	NM_173966	CACTCAGGTCCTCTTCTCA

### Effect of exogenous IFNG

bMDM were infected with *M*. *bovis* as described above. After 1 h the medium was removed and replaced with bMDM medium containing different concentrations of recombinant bovine IFNG (Bio-Rad).

### Quantitative RT-PCR (RT-qPCR) analysis

RNA was extracted from bMDM using the ReliaPrep RNA cell miniprep kit (Promega) with an on-column DNAse step as directed by the manufacturers. RNA was quantified using the NanoDrop ND-1000 spectrophotometer (Thermo Fisher Scientific). First strand cDNA was reverse transcribed from 200–500 ng total RNA using oligo(dT) primer and GoScript reverse transcriptase (Promega) according to the manufacturer’s instructions. The resulting cDNA was diluted 1:20 for all genes. Oligonucleotides were designed for each gene using Primer3 [15, 16] and Netprimer (Biosoft International) software (Table 2). The qPCR reaction was carried out as described previously [6]. The relative quantities of mRNA were calculated using the method described by Pfaffl (2001) [17], using the RT-qPCR results for RALBP1 associated Eps domain containing 1 (REPS1) to calculate differences in the template RNA levels and thereby standardize the results for the genes of interest.

**Table 2:** Details of the RT-qPCR oligonucleotides used in the study.

Gene	Accession No.	Orientation	Primer sequence (5’-3’)
interferon gamma (IFNG)	NM_174086	F R	GCAAGTCTATGGGATTTCAAGG GGCATCATTTCATTTATCAGCA
interferon gamma receptor 1 (IFNGR1)	NM_001035063	F R	CTTCTCTTTCTTGTATTTGCTCTGG ACTTGGTGCCTTCTCGCTTA
interleukin 1, beta (IL1B)	NM_174093	F R	TCCGACGAGTTTCTGTGTGA TGTGAGAGGAGGTGGAGAGC
interleukin 6 (IL6)	NM_173923	F R	ACCACTCCAGCCACAAACAC ATGCCCAGGAACTACCACAA
interleukin 10 (IL10)	NM_174088	F R	TGGATGACTTTAAGGGTTAC AGGGCAGAAAGCGATGAC
nitric oxide synthase 2 (NOS2/iNOS)	NM_001076799	F R	GAGGAGATGCTGGAGATGGC TGAACATAGACCTTGGGCTGG
tumour necrosis factor (TNF)	NM_173966	F R	GGGACACCCAGAATGTGAG ATTGGCAGGAAGGGAGAGTT
RALBP1 associated Eps domain containing 1 (REPS1)	NM_001193011	F R	AAGCCGAGAAACATCCAGAG ACATTGGCGGGAGCACTA

F and R denote forward and reverse primers respectively

### Enzyme‑linked immunosorbent assays (ELISA)

IL10, IL1B and IFNG protein levels were quantified in supernatants collected from bMDM. IL10 protein was quantified by ELISA using the mouse anti-bovine IL10 antibodies MCA2110 and MCA211b (Bio-Rad) as coating and detecting antibodies respectively. Both antibodies were used at 2 μg/ml final concentration. The concentration of IL10 was determined using a standard curve of recombinant ovine IL10 (kindly provided by Dr. Jayne Hope, The Roslin Institute). IL1B and IFNG protein levels in the supernatants were quantified using the Bovine IL1β Screening Kit (Thermo Scientific Pierce) and bovine IFNG specific ELISA Assay kit (Bio-Rad), respectively, following the manufacturers’ instructions. All ELISAs were carried out using Nunc-Immuno Maxisorp 96 well plates, with all samples and standards in triplicate. The plates were read at 450 nm, with the reference values at a wavelength of 550 nm subtracted, using a Cytation 3 plate reader (BioTek).

### Griess assay

The production of nitric oxide (NO) by bMDM infected with *M*. *bovis* was quantified by measuring nitrite in the supernatants using the Griess Reagent System (Promega) following the manufacturer’s instructions. Absorbance was measured at 540 nm using a Cytation 3 plate reader (BioTek).

### Statistical analysis

All statistical analysis was carried out using Minitab version 17. The data were transformed on the log_10_ scale before statistical analyses to stabilize the variance. Changes in gene expression in response to the two different *M*. *bovis* strains were analysed by t-test. Comparisons between bMDM treated with different siRNA were analysed by General Linear Model (GLM), fitting biological replicate as a random effect, with time (where applicable) and siRNA as fixed effects. Subsequent Fisher’s tests were used to identify significant differences between siRNA treatments, as well as the interaction between siRNA and time.

## Results

### Differential expression of IL10 by bMDM infected with different *M*. *bovis* strains

**Fig 1:**
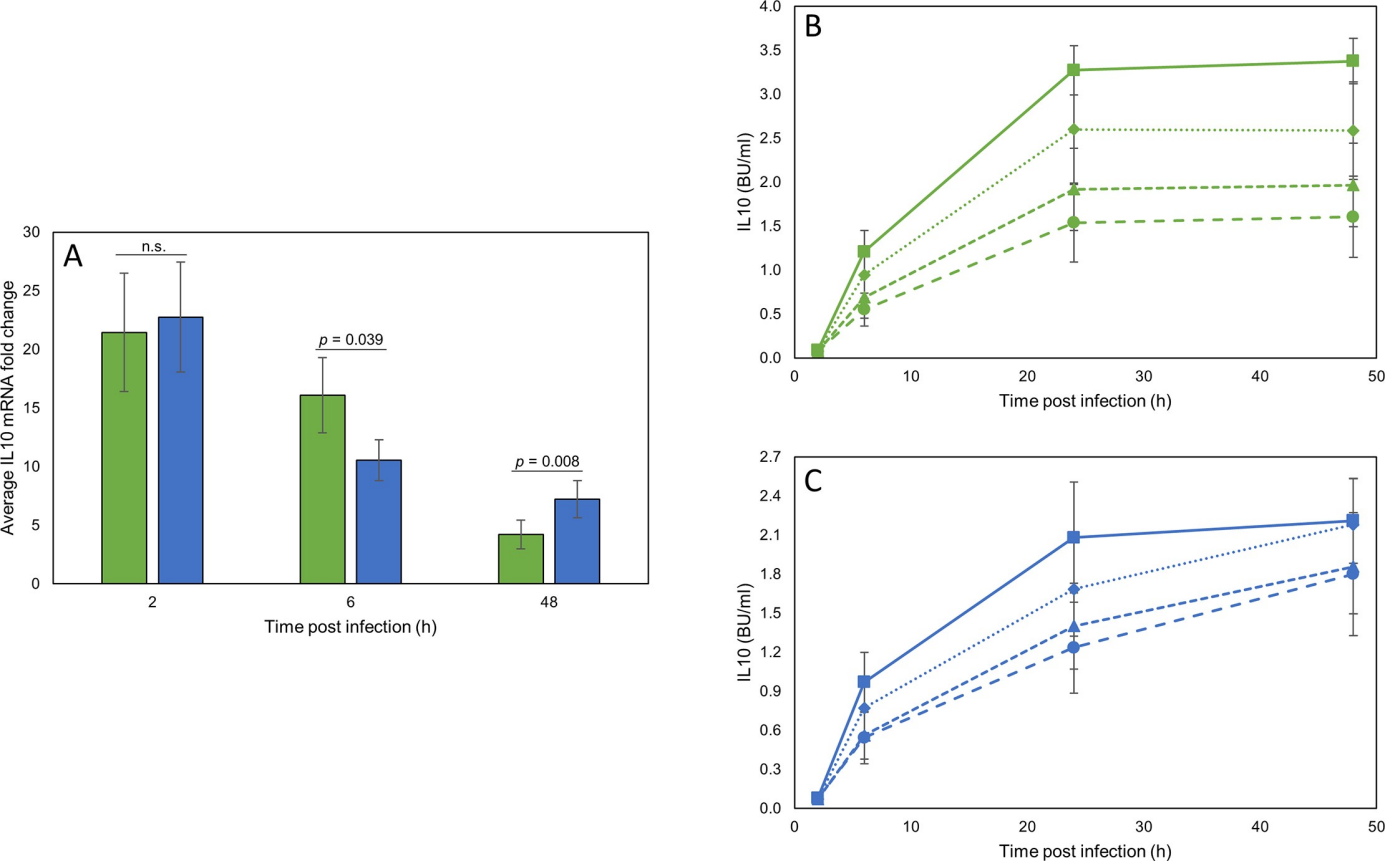
IL10 is differentially expressed by bMDM in response to infection with the two *M*. *bovis* strains and is regulated by IFNG. A) Summary of the RT-qPCR analysis of the average IL10 mRNA fold change during the infection of bMDM with *M*. *bovis* strains AF2122/97 (green bars) and G18 (blue bars) compared to uninfected controls. Error bars denote the standard error of seven biological replicates and the statistical significance is indicated on the figure, where n.s. denotes no significant difference. B & C) Graphs summarising the average IL10 protein production by bMDM infected with *M*. *bovis* strain AF2122/97 (B) and G18 (C) in the presence of different concentrations of exogenous recombinant bovine IFNG. BU denotes Biological Units. ■ denotes no exogenous IFNG, ◆ denotes 50 pg/ml IFNG, ▲ represents 500 pg/ml IFNG and ● denotes 5,000 pg/ml IFNG. Error bars denote the standard error of seven biological replicates.

Previously we have shown that significantly more IL10 mRNA was produced by bMDM at 24–72 hpi in response to infection with G18 than AF2122/97 [6]. To confirm this result the expression of IL10 was investigated in bMDM derived from a different cohort of animals. No significant difference in IL10 mRNA produced in response to bMDM infection with the two *M*. *bovis* strains was observed 2 hpi (Fig 1A). By 6 hpi IL10 transcription had decreased, but significantly more IL10 mRNA was detected in bMDM infected with AF2122/97 than G18, with on average 1.5 times more IL10 mRNA detected in AF2122/97 infected bMDM (*p* = 0.039), which was not observed in our previous study [6]. However, at 48 hpi the pattern of IL10 expression was reversed, with significantly more IL10 mRNA being produced by bMDM infected with G18 than AF2122/97. On average 2.2 times more IL10 mRNA was detected 48 hpi in bMDM infected with G18 than those infected with AF2122/97 (*p* = 0.008) (Fig 1A).

### Investigation of the mechanism underlying the *M*. *bovis* strain-specific differential expression of IL10 by bMDM

IL10 production is modulated by several mechanisms and we were interested to know what mechanism regulates the strain specific differential expression of IL10 during *M*. *bovis* infection. Production of IL10 is known to be inhibited by IFNG [18] and we have previously shown that IFNG is produced by bMDM infected with *M*. *bovis* and that significantly more was produced in response to AF2122/97 than G18 [6]. The differential production of IFNG by bMDM coincided with the divergence in IL10 mRNA transcription, with nearly twice as much IFNG protein being detected in supernatants 24 hpi from bMDM infected with AF2122/97 than from those infected with G18 [6]. Therefore, we postulated that IFNG was inhibiting IL10 production by AF2122/97 infected bMDM. To investigate this, we quantified IL10 protein production by bMDM during *M*. *bovis* infection in the presence of different concentrations of recombinant IFNG, which were added 1 hpi to prevent priming of bMDM. The results for AF2122/97 and G18 infected bMDM are summarised in Fig 1B and 1C, respectively. Due to the greater transcription of IL10 early in infection (Fig 1A), the amount of IL10 protein detected in supernatants was higher in response to infection with AF2122/97 than G18. However, the addition of exogenous IFNG significantly inhibited the production of IL10 protein in response to infection with both *M*. *bovis* strains. Average IL10 protein levels detected 24 h after AF2122/97 infection were reduced from 3.3 Biological Units (BU)/ml by untreated bMDM to 2.6 BU/ml (*p* = 0.026), 1.9 BU/ml (*p* < 0.001) and 1.5 BU/ml (*p* < 0.001) by bMDM treated with 50 pg/ml, 500 pg/ml and 5,000 pg/ml IFNG, respectively (Fig 1B). Similar results were observed during G18 infection. Although the reduction in IL10 protein from on average 2.1 BU/ml by untreated bMDM to 1.7 BU/ml in the presence of 50 pg/ml IFNG was not statistically significant (*p* = 0.238), there was a significant reduction in IL10 protein levels when bMDM were treated with 500 pg/ml and 5,000 pg/ml IFNG, with on average 1.4 BU/ml (*p* = 0.008) and 1.2 BU/ml (*p* = 0.002) IL10, respectively. These results support the hypothesis that differential expression of IFNG could regulate the *M*. *bovis* strain-specific differential expression of IL10.

### Investigating the importance of IL10 in determining the transcriptional response of bMDM to different *M*. *bovis* strains

Infection with the two *M*. *bovis* strains included in this study have previously been shown to induce a significantly different transcriptional response by bMDM [6]. The response to AF2122/97 was much greater, with respect to the number of differentially expressed genes and the degree of differential expression [6]. However, it was unclear if this variation was due to AF2122/97 inducing a hyper-response or G18 dampening down the response.

**Fig 2:**
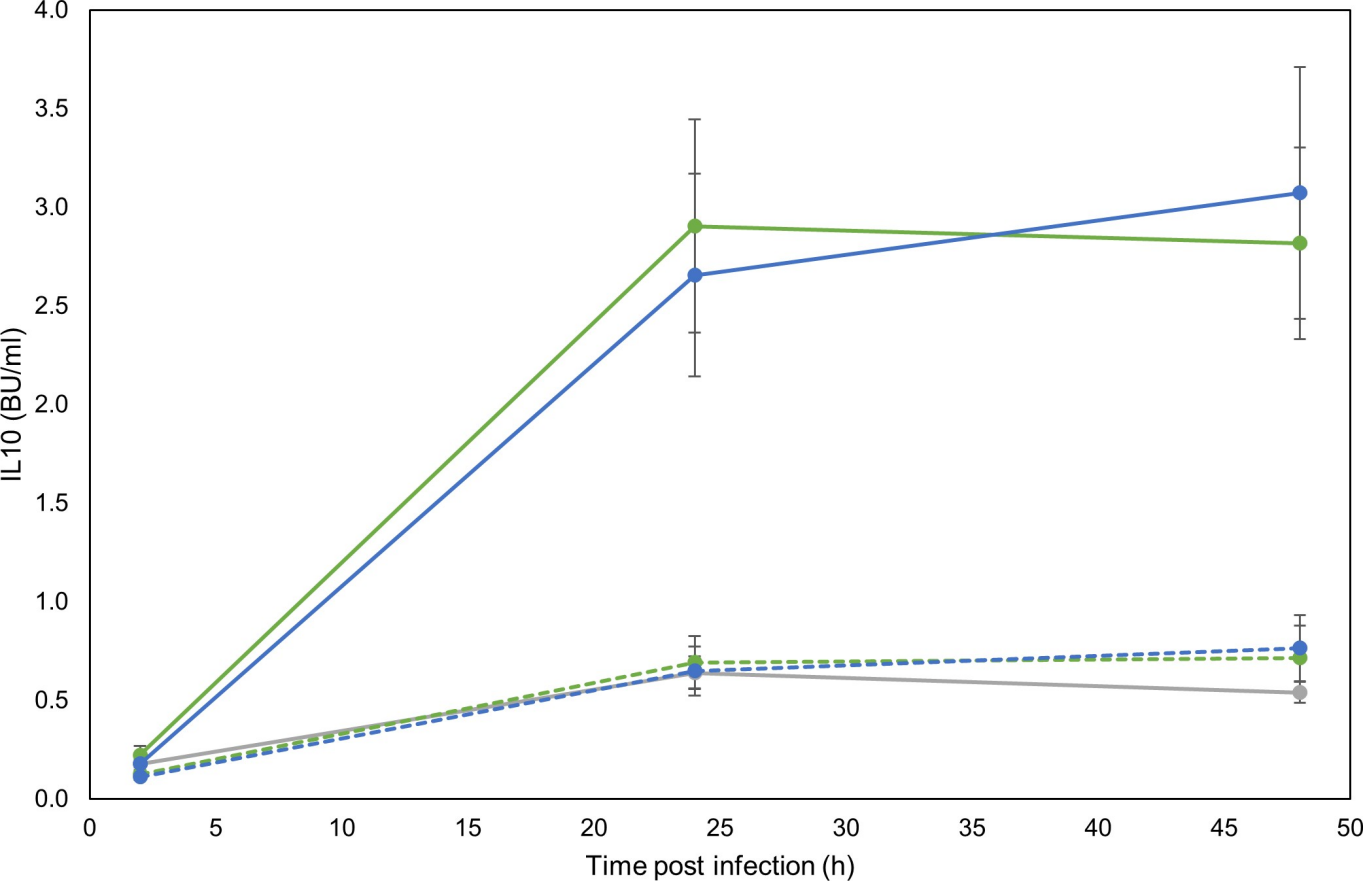
Successful knock-down of IL10 with siRNA. Graph illustrating successful knock-down by siRNA of IL10 protein production during infection with *M*. *bovis* strains AF2122/97 (green lines) and G18 (blue lines). Solid lines denote bMDM treated with NTC siRNA and dotted lines denote bMDM treated with IL10 siRNA 48 h prior to infection. The grey line represents IL10 production by uninfected bMDM. Error bars denote the standard error of seven biological replicates.

We hypothesized that the greater expression of IL10 in response to G18 infection may be a mechanism by which G18 silences the bMDM response to infection. To study this we investigated the effect of knocking-down the expression of IL10 by siRNA. There was no significant difference in cell viability of bMDM transfected with NTC siRNA or IL10 siRNA (S1 File). However, approximately 75% less IL10 protein was detected in supernatants from bMDM treated with IL10 siRNA compared to those treated with NTC siRNA (Fig 2). The IL10 protein levels were reduced to a level similar to that observed in supernatants from uninfected bMDM. There was no significant difference in the IL10 protein detected in the supernatants induced by the two *M*. *bovis* strains, which differs from earlier results (Fig 1B and 1C), but has been observed before [6] and relates to the accumulation of protein in the supernatants produced early in infection before the transcription of IL10 induced by the two *M*. *bovis* strains diverges.

**Fig 3:**
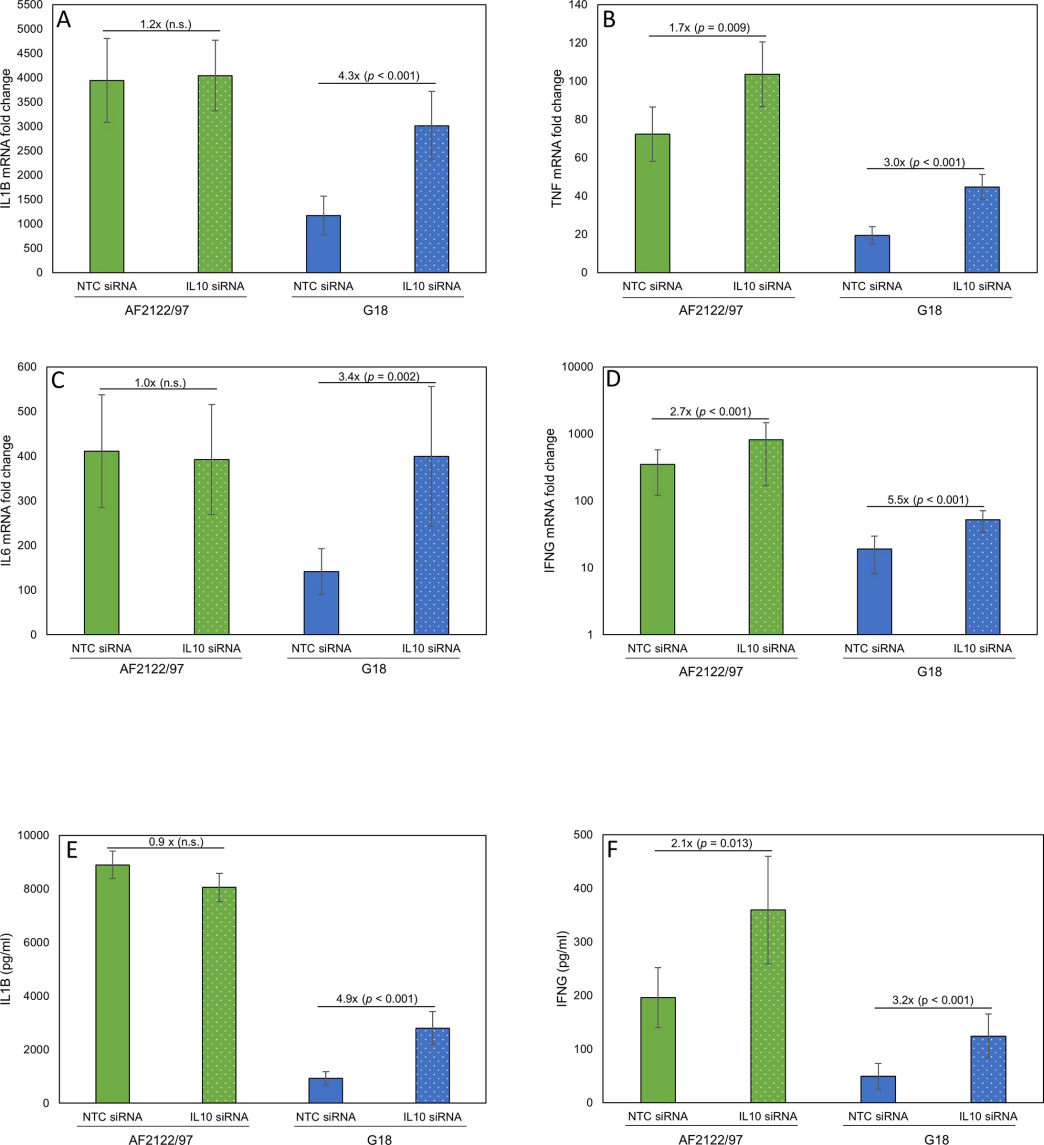
IL10 knock-down has variable effects on the cytokine response of bMDM to infection with *M*. *bovis* strains. Average A) IL1B, B) TNF, C) IL6 and D) IFNG mRNA fold change 24 hpi compared to uninfected controls. Average E) IL1B protein and F) IFNG protein detected in supernatants 48 hpi and 24 hpi, respectively. bMDM were infected with *M*. *bovis* strains AF2122/97 (green bars) or G18 (blue bars) 48 h after transfection with NTC siRNA (solid bars) or IL10 siRNA (dotted bars). Error bars denote the standard error of nine biological replicates. The average fold difference (IL10 siRNA compared to NTC siRNA) and the *p* value of the statistical analysis are indicated on the bars.

The effect of IL10 knock-down on the expression of a subset of pro-inflammatory cytokines; IL1B, IL6, TNF and IFNG, was quantified. All four investigated genes exhibited *M*. *bovis*-strain specific differential expression. On average 5.6 times more IL1B mRNA (*p* < 0.001), 5.2 times more IL6 mRNA (*p* < 0.001), 4.3 times more TNF mRNA (*p* < 0.001) and 48.5 times more IFNG mRNA (*p* < 0.001) were produced by bMDM pre-treated with NTC siRNA in response to AF2122/97 infection than G18 infection (Fig 3). The mRNA levels of all four genes were significantly higher 24 hpi in G18 infected bMDM in the absence of IL10 compared to NTC siRNA treated controls (Fig 3A–3D). For example, IFNG mRNA levels were on average 5.5 times higher in response to G18 infection when IL10 was knocked-down. IL10 knock-down resulted in the production of IL1B and IL6 mRNA levels similar to that produced by AF2122/97 infected bMDM treated with NTC siRNA (Fig 3A and 3C). However, levels of TNF and IFNG were still significantly lower than observed in AF2122/97-infected bMDM (Fig 3B and 3D). In contrast to the effect of IL10 knock-down during G18 infection of bMDM, IL1B and IL6 mRNA levels were not significantly affected by IL10 knock-down during AF2122/97 infection (Fig 3A and 3C). However, IFNG and TNF expression were significantly affected by the presence of IL10, with on average 2.7 and 1.7 times more IFNG and TNF mRNA, respectively, detected in bMDM pre-treated with IL10 siRNA compared to NTC siRNA (Fig 3B and 3D).

To confirm the biological effect of IL10 on the expression of other cytokines, the production of IL1B and IFNG protein was quantified by ELISA (Fig 3E and 3F). Previously we have shown that *M*. *bovis* infection results in the release of pro-IL1B and the active form, which are both detected in the ELISA [6]. In agreement with the RT-qPCR analysis (Fig 3A) the amount of IL1B protein secreted by G18 infected bMDM were significantly higher when IL10 was reduced, with on average 4.9 times more IL1B being detected in the supernatants (Fig 3E). However, the production of IL1B was not affected by IL10 knock-down during AF2122/97 infection at the mRNA or protein level (Fig 3E). In contrast, IFNG produced by bMDM in response to AF2122/97 and G18 infection increased when IL10 was knocked-down (Fig 3F). On average 2.1 and 3.2 times more IFNG were produced by bMDM treated with IL10 siRNA rather than NTC siRNA during AF2122/97 and G18 infection, respectively.

The results reveal that IL10 has variable effects during infection of bMDM with different *M*. *bovis* strains. During G18 infection the expression of all investigated proinflammatory cytokines was modulated by IL10. The action of IL10 appears to be solely responsible for the suppression of IL1B and IL6 production during G18 infection, thus supporting our hypothesis that IL10 induced during G18 infection is dampening down some of the transcriptional response of bMDM to infection. However, other aspects of the strain-specific differential transcriptional response of bMDM are being regulated by additional, currently unidentified, mechanisms.

**Fig 4:**
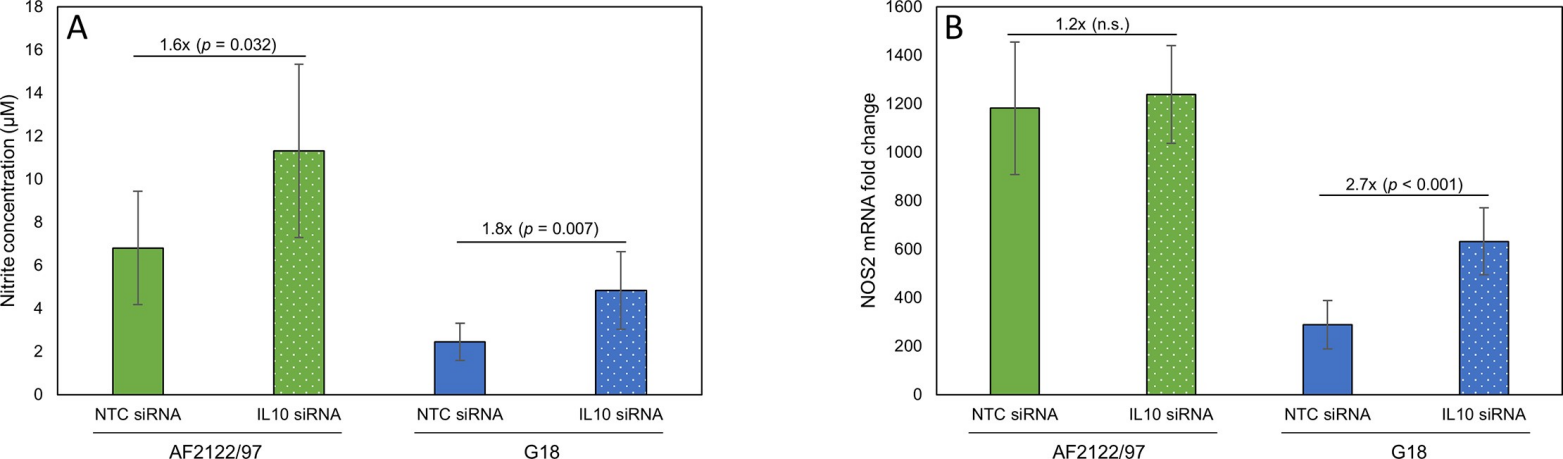
IL10 regulates nitric oxide production during *M*. *bovis* infection. A) Average nitrite levels in supernatants 48 hpi and B) average NOS2 mRNA fold change 24 hpi for bMDM infected with *M*. *bovis* strains AF2122/97 (green bars) or G18 (blue bars). bMDM were treated with NTC siRNA (solid bars) or IL10 siRNA (dotted bars) 48 h prior to infection. Error bars denote the standard error of nine biological replicates. The average fold difference (IL10 siRNA compared to NTC siRNA) and the *p* value of the statistical analysis are indicated on the bars.

The effect of IL10 on the production of nitric oxide (NO) was also investigated by quantifying the amount of the breakdown product nitrite (NO_2_^-^) in the supernatants. Significantly more NO_2_^-^ was detected in response to AF2122/97 than G18 infection (*p* = 0.026). In addition, the amount of NO_2_^-^ was significantly higher in bMDM treated with IL10 siRNA, with on average 1.6 and 1.8 times more NO_2_^-^ produced by AF2122/97 and G18 infected bMDM, respectively (Fig 4A). The production of NO during Mø activation is mainly regulated by modifying transcription of nitric oxide synthase 2 (NOS2/iNOS) (reviewed by [19]). To investigate if NO production was being regulated at the transcriptional level, the amount of NOS2 mRNA was quantified. As with the investigated pro-inflammatory cytokines, NOS2 mRNA levels were significantly higher, with on average 2.7 times more NOS2 mRNA (*p* < 0.001) being detected, during G18 infection in the absence of IL10 (Fig 4B). However, NOS2 mRNA levels were not significantly affected during AF2122/97 infection by the absence of IL10. Therefore, IL10 appears to regulate NO production by different mechanisms during AF2122/97 and G18 infections.

### Effect of IL10 on *M*. *bovis* intracellular survival

**Fig 5:**
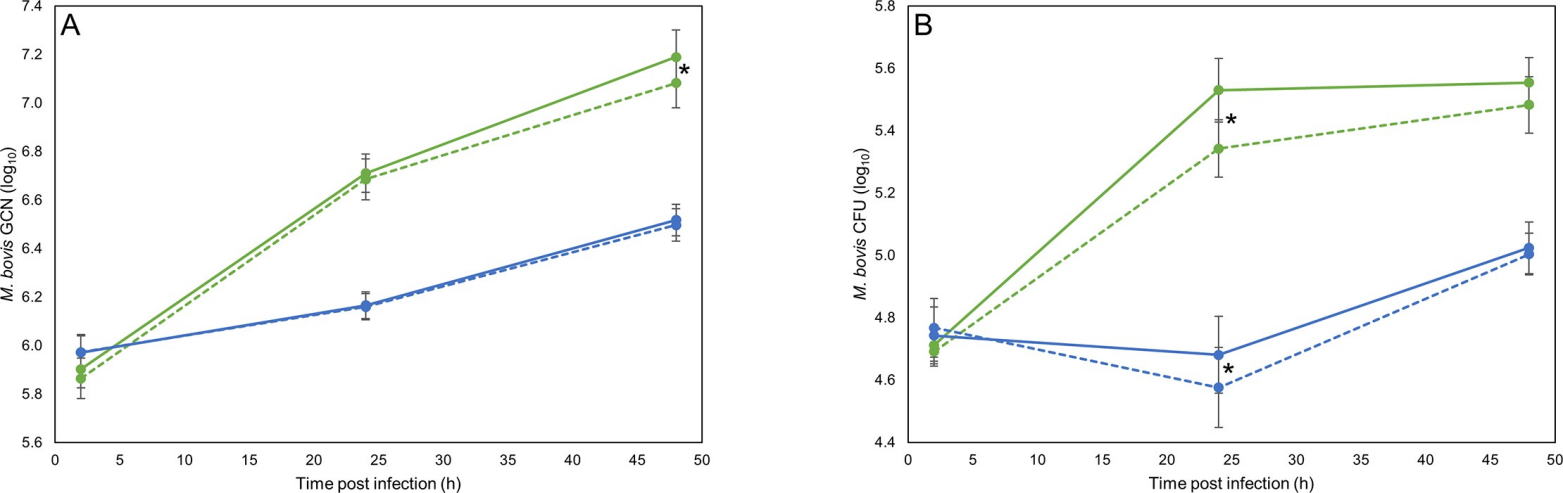
IL10 promotes survival of *M*. *bovis* during bMDM infection. Graphs summarising the average number of intracellular *M*. *bovis* AF2122/97 (green lines) and G18 (blue lines) during bMDM infection quantified by A) GCN and B) CFU counts. bMDM were treated 48 h prior to infection with NTC siRNA (solid lines) or IL10 siRNA (dotted lines). Error bars denote the standard error of seven biological replicates. * denotes that the siRNA had a statistically significant effect on intracellular *M*. *bovis* numbers by GLM and subsequent Fisher’s test (*p* < 0.05).

The results described above suggest that the suppression of IL10 has a greater effect on the bMDM response to G18 infection than AF2122/97 infection, supporting the hypothesis that IL10 is more important for promoting infection of G18 than AF2122/97. Therefore, the effect of IL10 on the survival and/or replication of intracellular *M*. *bovis* was investigated. Intracellular mycobacteria numbers were quantified by two methods; GCN, which detects live and non-viable mycobacteria, and CFU, which quantifies viable mycobacteria. Knocking-down IL10 had no effect on mycobacterial uptake by bMDM and, as observed previously [6], similar numbers of intracellular G18 and AF2122/97 were detected 2 hpi by GCN (Fig 5A) and CFU (Fig 5B). However, thereafter the growth of the two *M*. *bovis* strains differed significantly, as observed previously [6], with much greater growth of AF2122/97, especially during the first 24 h. In contrast, numbers of G18 measured by CFU dropped during the first 24 h (Fig 5B), although the GCN quantification illustrates that replication was occurring at a low level during this time (Fig 5A). The mechanism behind this profound difference in early intracellular survival is currently unknown, but presumably is due to genetic differences between the *M*. *bovis* strains, which may affect how they modulate the microenvironment of the phagosome they dwell in.

In disagreement with our hypothesis, the loss of IL10 only caused a significant difference in the GCN of AF2122/97 at 48 hpi (Fig 5A), with on average 19% less *M*. *bovis* genomes being detected in IL10 siRNA treated bMDM (*p* = 0.019). However, a significant reduction in CFU numbers was detected for both AF2122/97 and G18 at 24 hpi (Fig 5B), with on average a 32% (*p* = 0.013) and 21% (*p* = 0.044) reduction in intracellular AF2122/97 and G18, respectively. This suggests that even though IL10 knock-down had a greater effect on the bMDM response to G18 infection, IL10 is important for the survival of both *M*. *bovis* strains during bMDM infection. In fact, the effect of IL10 knock-down appears to be more transient in G18 infection, as no effect on bacteria numbers was observed at 48 hpi.

### Investigation of the mechanism(s) behind the IL10 effect on *M*. *bovis* survival/growth in bMDM

**Fig 6:**
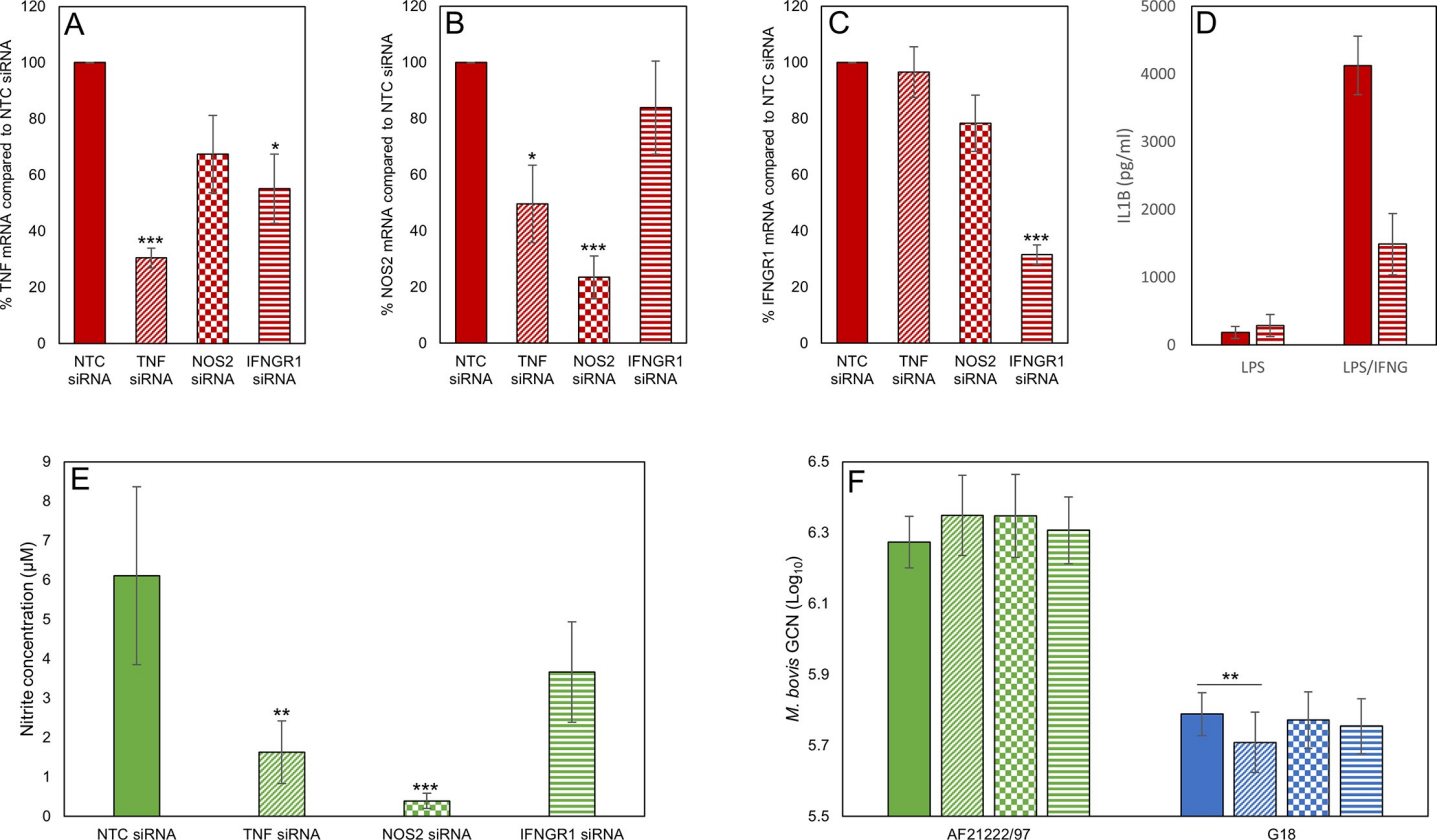
Regulation of TNF, IFNG and NO production do not individually account for the effect of IL10 on intracellular *M*. *bovis* survival. Graphs confirming A) TNF, B) NOS2 and C) IFNGR1 gene knock-down 24 h post LPS activation of bMDM pre-treated with NTC siRNA or siRNA targeting TNF, NOS2 and IFNGR1 quantified by RT-qPCR. The results are expressed as the percentage mRNA compared to the NTC siRNA sample. Error bars denote the standard error of five biological replicates. D) Confirmation that IFNGR1 knock-down inhibits IFNG signalling by illustrating the production of IL1B by bMDM pre-treated with NTC siRNA (solid bars) or IFNGR1 siRNA (striped bars) 24 h post activation with LPS with/without recombinant IFNG. Error bars denote the standard deviation of two biological replicates. E) Graph summarizing the effect of target gene knock-down on NO production quantified by measuring the nitrite concentration in the supernatants 48 hpi with *M*. *bovis* strain AF2122/97. Error bars denote the standard error of five biological replicates. F) Graph summarizing the average number of intracellular *M*. *bovis* AF2122/97 (green bars) and G18 (blue bars) quantified by GCN at 24 hpi. bMDM were treated 48 h prior to infection with NTC siRNA (solid bars) or siRNA targeting TNF (diagonal striped bars), NOS2 (checker-board bars) and IFNGR1 (horizontal striped bars). Error bars denote the standard error of five biological replicates. * denotes *p* < 0.05, ** denotes *p* < 0.005 and *** denotes *p* < 0.001 statistically significant effect of siRNA compared to NTC siRNA by GLM and subsequent Fisher’s test.

Reducing IL10 expression in bMDM during *M*. *bovis* infection affected the survival and/or replication of both AF2122/97 and G18. We predicted that the mechanism behind this effect of IL10 is likely to be conserved between *M*. *bovis* strains. Our investigations on the transcriptional response of bMDM found that the production of TNF and IFNG in response to infection with both *M*. *bovis* strains was affected by removing IL10. Furthermore, the NO response was also affected with both strains, although possibly by different mechanisms. Therefore, we wished to investigate if these three down-stream effects of IL10 were responsible for IL10 promoting *M*. *bovis* infection. This was addressed using siRNA targeting TNF, NOS2 and interferon gamma receptor 1 (IFNGR1/IFNGRα). The TNF and NOS2 mRNA levels were reduced by approximately 70% and 77%, respectively, with the relevant target siRNA (Fig 6A and 6B). Unfortunately, attempts to directly knock-down IFNG mRNA levels failed and therefore we targeted a component of the IFNG receptor, IFNGR1. This resulted in an approximately 68% reduction in IFNGR1 mRNA levels (Fig 6C), which reduced IFNG signalling by approximately two-thirds (Fig 6D). Knocking-down NOS2 reduced the nitrite levels in the supernatants 48 hpi by 93% with AF2122/97 infection (Fig 6E). Knocking-down TNF also significantly reduced NO production by 73% (Fig 6E), by indirectly reducing NOS2 mRNA levels (Fig 6B), illustrating the complexity of the bMDM response to *M*. *bovis* infection.

Pre-treatment with the different siRNA had no effect on *M*. *bovis* GCN 2 hpi (S1 Fig). Furthermore, knock-down of these three target genes individually had no significant effect on the survival and/or growth of AF2122/97 at 24 hpi or 48 hpi, quantified by GCN (*p* > 0.05) (Fig 6F and S1 Fig). However, it should be noted that in four out of the five biological replicates knocking-down TNF and NOS2 resulted in higher AF2122/97 GCN 24 hpi. Therefore, host genetics may play a role in the importance of these genes during infection. Reducing TNF production had a significant effect on intracellular G18 survival in bMDM (Fig 6F). Unexpectedly, knocking-down TNF was detrimental to G18 survival in bMDM, with a 16% reduction in intracellular G18 at 24 hpi (*p* = 0.003) (Fig 6D). However, this effect was lost by 48 hpi. NOS2 and IFNGR1 knock-down had no significant effect on G18 GCN at 24 hpi (Fig 6F) or 48 hpi (S1 Fig). Overall, the results suggest that alternative mechanisms are involved in IL10 promoting *M*. *bovis* infection of bMDM.

## Discussion

IL10 is an important regulator of the immune response, dampening down its activity to limit immune-associated pathology. A range of pathogens have evolved different ways to manipulate IL10 signalling to promote their survival (reviewed by [7, 20]). Pathogenic mycobacteria species use several mechanisms to augment IL10 production. *M*. *tuberculosis* and MAP modulate the balance of histone deacetylases that alter the chromatin conformation at the IL10 promoter, thus allowing greater access by transcription factors [12, 21]. Furthermore, MAP down-regulates production by murine Mø of a miRNA, miRNA-27a-3p, that indirectly inhibits IL10 [11]. The fact that mycobacteria use multiple mechanisms to enhance IL10 production supports the hypothesis that this cytokine plays an important role in the interaction between host Mø and pathogens. This has been confirmed by several studies investigating the effect of reducing IL10 expression during infection with MAP [9], *M*. *bovis* [10] and *M*. *tuberculosis* [8].

Our interest in IL10 arose from the discovery that two strains of *M*. *bovis* induced differential expression of IL10 by bMDM, which correlated with the intensity of the bMDM response to infection. The *M*. *bovis* strain G18, which induced prolonged expression of IL10, elicited a more silent and less cytotoxic infection than the *M*. *bovis* strain AF2122/97 [6]. We wished to investigate if IL10 was involved in dampening down the response of bMDM to G18. Firstly, we investigated the cause of the differential expression of IL10. As a master regulator of the immune response, IL10 production is regulated at multiple stages, including chromatin remodelling and post-transcriptional regulation (reviewed by [18, 22]). We identified IFNG as an inhibitor of IL10 production during *M*. *bovis* infection of bMDM. IFNG inhibits MAP kinases and regulates GSK3 to suppress the transcription factors CREB and AP-1, which positively regulate IL10 expression in human Mø [23]. Further work is required to investigate if this is the mechanism involved in IFNG suppression of IL10 production during *M*. *bovis* infection.

Reducing IL10 production by bMDM with siRNA reduced intracellular *M*. *bovis* numbers by over twenty percent at 24 hpi measured by CFU. CFU quantifies live *M*. *bovis* and no significant difference was observed by quantifying GCN at this time point, which quantifies live and inactive *M*. *bovis*. Therefore, *M*. *bovis* replication was occurring, but more mycobacteria were being killed in the absence of IL10. The use of IL10 siRNA revealed that bMDM-derived IL10 was of similar importance for the survival of G18 and AF2122/97, in contrast to what we had hypothesized. There was no significant difference in the percentage reduction in G18 and AF2122/97 CFU quantified 24 hpi. Furthermore, no IL10 associated difference in *M*. *bovis* CFU was observed 48 hpi, although there was a reduction in AF2122/97 GCN at this time point. Therefore, the effect of IL10 was transient and was overcome by G18 and, to a lesser extent, AF2122/97. This suggests that the early production of IL10, when there was no significant difference in the amount produced by bMDM in response to AF2122/97 and G18 [6], is most important for *M*. *bovis* survival, not IL10 production after 24 h when the strain-specific differential expression of IL10 was observed. The biological relevance of this transient effect of IL10 on intracellular *M*. *bovis* is unclear and could only be resolved in the context of *in vivo* infections involving the entire immune system. The transient effect of IL10 on *M*. *bovis* intracellular growth described here differs from a previous study which found that the addition of anti-IL10 antibody resulted in a significant reduction in *M*. *bovis* replication five days post infection [10]. This discrepancy may relate to differences in the experimental design of the two studies. The earlier study used bMDM primed with LPS and IFNG before infection, used a lower MOI and reduced IL10 levels using a neutralizing antibody.

AF2122/97 infection of bMDM induces a much greater transcriptional response than G18, including a multitude of pro-inflammatory mediators, e.g. cytokines. The role of IL10 in regulating the transcriptional response of bMDM to the two *M*. *bovis* strains was therefore investigated. Blocking IL10 has been shown to increase TNF, IL12 and IL8 production by bMDM [9] and enhance IFNG production by bovine PBMC [24] infected with MAP. Similarly, treatment of bMDM with IL10 siRNA enhanced expression of IL1B, TNF, IL6, IFNG and NOS2 induced by G18 infection. Therefore, IL10 is involved in regulating the transcriptional response of bMDM to *M*. *bovis* infection. However, this effect was strain specific, with only TNF and IFNG mRNA levels being augmented by IL10 siRNA during AF2122/97 infection. This highlights the importance of confirming observations with more than one *M*. *bovis* strain. Although AF2122/97 is widely considered as a UK reference strain of *M*. *bovis* and frequently used in experimental infection studies [25, 26], it appears to differ significantly from G18 and therefore may not be representative of the *M*. *bovis* strains currently circulating in the UK and globally.

IL1B and IL6 expression by AF2122/97 infected bMDM was not affected by blocking IL10 production. Interestingly, blocking IL10 during G18 infection resulted in elevated expression of these two cytokines to similar levels measured during AF2122/97 infection. Therefore, the *M*. *bovis* induced production of IL10 is solely responsible for the strain-specific differential expression of these genes. However, although IL10 siRNA resulted in elevated TNF and IFNG mRNA levels during G18 infection, they remained significantly lower than that measured during AF2122/97 infection. Therefore, other, currently unknown, factors are co-regulating the expression of these genes.

The work described here shows that IL10 is involved in the regulation of NO production by bMDM during *M*. *bovis* infection. However, the mechanism involved may differ with different *M*. *bovis* strains. Levels of the NO breakdown product nitrite in the supernatants were significantly higher when IL10 was knocked-down during bMDM infection with both *M*. *bovis* strains. Regulation of NOS2 expression is the principal mechanism controlling NO production in immune cells (reviewed by [19]). However, NOS2 mRNA levels were only affected by IL10 siRNA during G18 infection. Therefore, NO production in AF2122/97 infected bMDM must be regulated by another mechanism. Two other genes encode NO synthases that can synthesize NO; NOS1 and NOS3. These are expressed at extremely low levels in bMDM and mRNA levels were not affected by *M*. *bovis* infection [6]. This suggests that NOS2 is being regulated at the post-translational or enzymatic level during AF2122/97 infection. The availability of arginine, the precursor of NO, is the rate-limiting step for NOS2 activity and can be modulated by changes in arginine transport or competition with other biochemical pathways. In particular, arginases can down-regulate NO production by competing with NOS2 for arginine (reviewed by [27]). Interestingly, arginase 2 is up-regulated in bMDM to a greater extent and for a longer duration during infection with AF2122/97 than G18 [6]. Further work is required to ascertain the mechanism behind the regulation of NO production by IL10 during AF2122/97 infection.

In this study we investigated if the regulation of TNF, IFNG and NO by IL10 was important in promoting *M*. *bovis* survival. Previous work supports this theory, for example NOS2 deficient mice are highly susceptible to *M*. *tuberculosis* infection [28]. However, the importance of NO during *M*. *bovis* infection is less clear. Treatment of bMDM with the NO inhibitor n^G^-monomethyl-L-arginine monoacetate (MMLA) enhanced intracellular growth of *M*. *bovis* in one study [29], whilst MMLA had no effect in an earlier study [10], in agreement with our results. The disparity may be due to the different strains of *M*. *bovis* investigated in these studies, significantly less NO was generated by bMDM in response to G18 than AF2122/97. The importance of TNF during mycobacterial infections has been illustrated in mouse studies. It plays a pivotal role in the maintenance of the structure and function of the granuloma, but also acts by inducing the anti-mycobacterial activity of murine Mø via inducing reactive nitrogen intermediates and host cell apoptosis (reviewed by [30, 31]). However, TNF is a pleiotropic cytokine and the relative abundance of TNF can determine whether it is protective or deleterious. TNF has been shown to promote growth of virulent *M*. *tuberculosis* in human alveolar Mø [32]. This agrees with our finding that pre-treatment of bMDM with siRNA against TNF was deleterious for the intracellular growth of *M*. *bovis* strain G18 (Fig 6F), but interestingly not AF2122/97.

Individually knocking-down the IL10-target genes TNF, NOS2 and IFNG did not promote *M*. *bovis* growth/survival in bMDM. It is possible that the incomplete silencing of these genes by siRNA failed to reduce their levels to below that inducing a biological effect. Alternatively, IL10 suppression of these targets may synergistically affect *M*. *bovis* survival, which would require all three genes to be knocked-down simultaneously to observe an effect. However, the expression of these genes is interlinked, illustrated by the fact that knock-down of each one altered the expression of at least one of the others (Fig 6), resulting in, at least partial, simultaneous knock-down of the genes. Therefore, we conclude that IL10 promotes *M*. *bovis* growth/survival in bMDM by a mechanism other than inhibiting the production of IFNG, TNF and NO.

Our *M*. *bovis* survival studies suggest that IL10 expression during the first 24 h of infection is important for promoting *M*. *bovis* survival in bMDM. Therefore, an alternative mechanism by which IL10 may promote *M*. *bovis* survival in bMDM may relate to the ability of IL10 to manipulate phagosome maturation. Upon infection *M*. *tuberculosis* inhibits the maturation of the phagosome it resides within, preventing phagosome-lysosome fusion which would result in mycobacterial degradation [33]. This inhibition requires live *M*. *tuberculosis* and IL10 [8]. Treatment of human and bovine Mø with neutralizing anti-IL10 antibody increased the acidification and maturation of mycobacteria containing phagosomes and reduced intracellular *M*. *tuberculosis* and MAP growth, respectively [8, 9]. Alternatively, intracellular survival of *M*. *tuberculosis* has been associated with the inhibition of Mø autophagy, through the activity of the protein Enhanced Intracellular Survival (EIS), which depends on the production of IL10 [21]. This protein is also encoded in the *M*. *bovis* genome and may therefore function similarly in bMDM.

In conclusion, we have furthered our understanding of the interaction of two strains of *M*. *bovis* with the infected Mø by investigating the role of IL10 during infection of bMDM. Our experiments addressed three main questions. We have identified IFNG as a regulator of IL10 production during *M*. *bovis* infection, which could account for the differential expression of IL10 observed in response to the two strains of *M*. *bovis*. Furthermore, we have shown the IL10 is responsible for the ability of the *M*. *bovis* strain G18 to dampen down at least part of the transcriptional response of bMDM to infection. However, additional, currently unknown, pathways must be involved in the strain-specific differential transcriptional response of bMDM. Finally, even though IL10 was differentially expressed in response to the two *M*. *bovis* strains, this cytokine was found to be equally important in promoting *M*. *bovis* survival during the early stage of infection and treatment of cells with siRNA targeting IL10 resulted in a significant reduction in intracellular *M*. *bovis* numbers. However, the *M*. *bovis* strain specific differential effects of IL10 on the response of bMDM to infection may have profound effects on the course of *in vivo* infection and the progression of bovine TB.

## Supporting information

S1 File Investigation of the effect of siRNA on cell viability. A description of the methodologies used to investigate the effect of siRNA on bMDM viability.
(PDF)

S1 Fig Effect of TNF, NOS2 and IFNGR1 knock-down on intracellular *M*. *bovis* survival. Graphs summarizing the average number of intracellular *M*. *bovis* AF2122/97 (green bars) and G18 (blue bars) quantified by GCN at A) 2 hpi and B) 48 hpi. bMDM were treated 48 h prior to infection with NTC siRNA (solid bars) or siRNA targeting TNF (diagonal striped bars), NOS2 (checker-board bars) and IFNGR1 (horizontal striped bars). Error bars denote the standard error of five biological replicates. Statistical analysis by GLM and subsequent Fisher’s test did not identify any significant effects of siRNA at either time point.
(TIF)
