# Supplementary material for: Interleukin 10 knock-down in bovine monocyte-derived macrophages has distinct effects during infection with two divergent strains of Mycobacterium bovis
Source: PLoS One. 2019 Sep 17;14(9):e0222437. doi: 10.1371/journal.pone.0222437 (PMC6748433; doi:10.1371/journal.pone.0222437)
Supplement: S1 File — A description of the methodologies used to investigate the effect of siRNA on bMDM viability. (PDF) [file pone.0222437.s001.pdf]

S1 File. Investigation of the effect of siRNA on cell viability

The transfection of siRNA into bovine monocyte derived macrophages (bMDM) has previously been optimized by our group [1]. Prior to use the effect of each siRNA on cell viability was investigated by studying the gross morphology of the cells by light microscopy and by comparing the concentration of RNA recovered from treated bMDM. Furthermore, cell viability was analysed by flow cytometry. Twelve-day old bMDM were transfected with siRNA as described previously [2]. After 24 h bMDM were harvested using TrypLE Express (Invitrogen) and treated with Zombie Aqua viability dye (BioLegend) following the manufacturer's instructions. The dye is non-permeant to live cells but taken up by cells with compromised membranes. bMDM were stained with anti-bovine CD172A conjugated with AF647 (Bio-Rad) before being fixed with 2% paraformaldehyde. Flow cytometry analysis was carried out using a BD LSRFortessa X20 (BD Biosciences) and the data was analysed using FCS Express 6. Fig. S1 summarizes the results of a typical experiment investigating the effect of non-target control (NTC) and IL10 siRNA on bMDM viability. Cells were initially gated to eliminate debris (A) and doublets (B). bMDM were unstained (C) or stained with Zombie Aqua and anti-bovine CD172A (D-F). bMDM were untreated with siRNA and transfection reagent (D), transfected with NTC siRNA (E) or IL10 siRNA (F). The results show similar levels of cell viability across the treatments.

Fig. S1

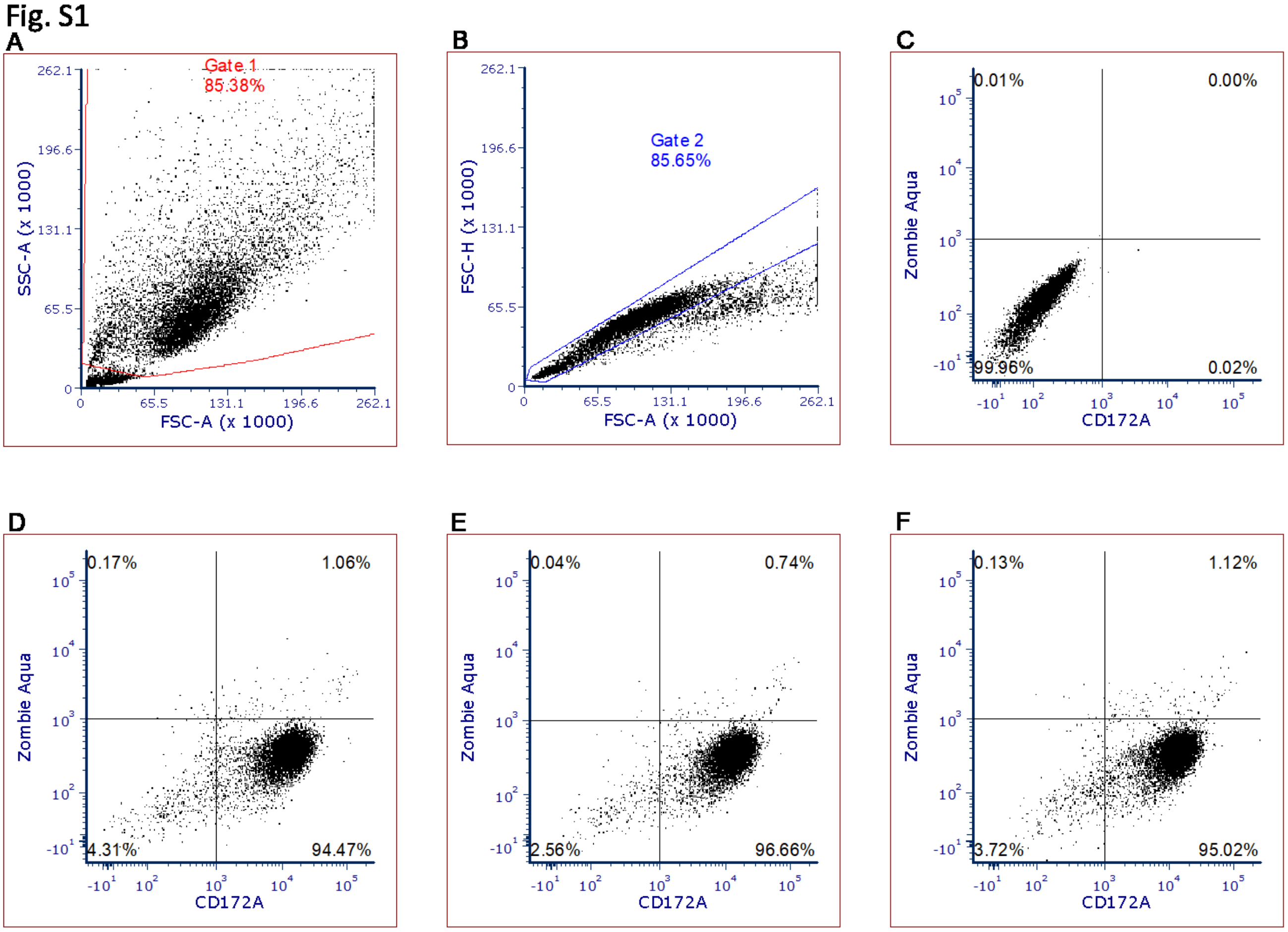

To further ensure that cell viability is not affected by siRNA treatment during infections, the concentration of RNA recovered from bMDM was compared. RNA was isolated using the ReliaPrep RNA cell miniprep kit (Promega) with an on-column DNase step as directed by the manufacturers. The resulting RNA was eluted in a constant volume of 30 µl across all samples and the concentration was measured using a Nanodrop ND-1000 spectrophotometer (Thermo Scientific). Fig. S2 summarizes the average concentration of RNA recovered from bMDM 24 h post infection with *M. bovis* strain AF2122/97 in samples without siRNA treatment (NC) and those treated with NTC siRNA or IL10 siRNA. There was no significant difference in the RNA concentration across the samples (t test, $p > 0.05$), suggesting a similar level of cell viability.

Fig. S2

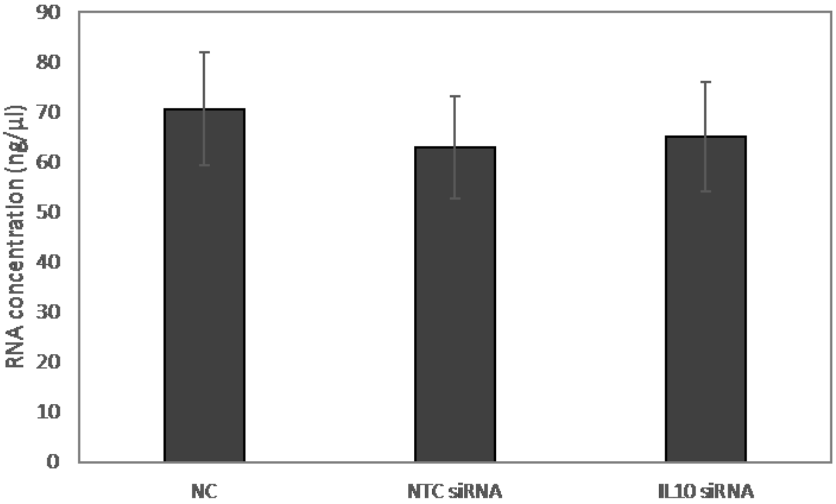

References

1. Jensen K, Anderson JA, Glass EJ. Comparison of small interfering RNA (siRNA) delivery into bovine monocyte-derived macrophages by transfection and electroporation. Vet Immunol Immunopathol. 2014;158: 224-232.

2. Jensen K, Gallagher IJ, Kaliszewska A, Zhang C, Abejide O, Gallagher MP *et al.* Live and inactivated *Salmonella enterica* serovar Typhimurium stimulate distinct transcriptome profiles in bovine macrophages and dendritic cells. Vet Res. 2016;47: 46.
