# Supplementary figures and images for: Interleukin 10 knock-down in bovine monocyte-derived macrophages has distinct effects during infection with two divergent strains of Mycobacterium bovis
Source: PLoS One. 2019 Sep 17;14(9):e0222437. doi: 10.1371/journal.pone.0222437 (PMC6748433; doi:10.1371/journal.pone.0222437)

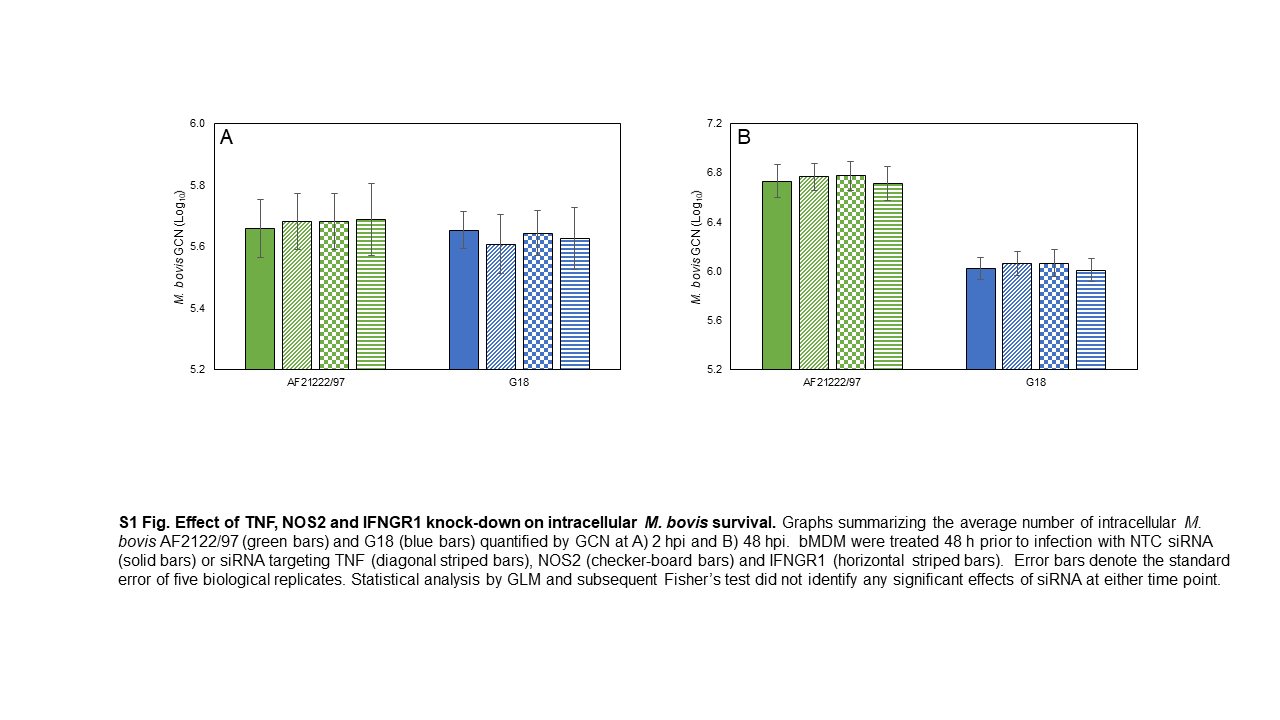

Supplement: S1 Fig — Graphs summarizing the average number of intracellular M. bovis AF2122/97 (green bars) and G18 (blue bars) quantified by GCN at A) 2 hpi and B) 48 hpi. bMDM were treated 48 h prior to infection with NTC siRNA (solid bars) or siRNA targeting TNF (diagonal striped bars), NOS2 (checker-board bars) and IFNGR1 (horizontal striped bars). Error bars denote the standard error of five biological replicates. Statistical analysis by GLM and subsequent Fisher’s test did not identify any significant effects of siRNA at either time point. (TIF) [file pone.0222437.s002.tif]
